# Regional differences in nutrient‐induced secretion of gut serotonin

**DOI:** 10.14814/phy2.13199

**Published:** 2017-03-21

**Authors:** Alyce M. Martin, Amanda L. Lumsden, Richard L. Young, Claire F. Jessup, Nick J. Spencer, Damien J. Keating

**Affiliations:** ^1^ Department of Human Physiology and Centre for NeuroscienceFlinders UniversityAdelaideAustralia; ^2^ South Australian Health and Medical Research Institute (SAHMRI)AdelaideAustralia; ^3^ Department of Anatomy and Histology and Centre for NeuroscienceFlinders UniversityAdelaideAustralia; ^4^ Adelaide Medical SchoolUniversity of AdelaideAdelaideAustralia

**Keywords:** Enterochromaffin, nutrients, serotonin

## Abstract

Enterochromaffin (EC) cells located in the gastrointestinal (GI) tract provide the vast majority of serotonin (5‐HT) in the body and constitute half of all enteroendocrine cells. EC cells respond to an array of stimuli, including various ingested nutrients. Ensuing 5‐HT release from these cells plays a diverse role in regulating gut motility as well as other important responses to nutrient ingestion such as glucose absorption and fluid balance. Recent data also highlight the role of peripheral 5‐HT in various pathways related to metabolic control. Details related to the manner by which EC cells respond to ingested nutrients are scarce and as that the nutrient environment changes along the length of the gut, it is unknown whether the response of EC cells to nutrients is dependent on their GI location. The aim of the present study was to identify whether regional differences in nutrient sensing capability exist in mouse EC cells. We isolated mouse EC cells from duodenum and colon to demonstrate differential responses to sugars depending on location. Measurements of intracellular calcium concentration and 5‐HT secretion demonstrated that colonic EC cells are more sensitive to glucose, while duodenal EC cells are more sensitive to fructose and sucrose. Short‐chain fatty acids (SCFAs), which are predominantly synthesized by intestinal bacteria, have been previously associated with an increase in circulating 5‐HT; however, we find that SCFAs do not acutely stimulate EC cell 5‐HT release. Thus, we highlight that EC cell physiology is dictated by regional location within the GI tract, and identify differences in the regional responsiveness of EC cells to dietary sugars.

## Introduction

Enteroendocrine (EE) cells are specialized epithelial cells within the gastrointestinal (GI) tract equipped to release an array of hormones and peptides in response to environmental cues. Collectively they constitute the largest endocrine tissue in our body. Enterochromaffin (EC) cells represent around half of all EE cells, and produce ~95% of total body serotonin (5‐hydroxytryptophan, 5‐HT), including all plasma 5‐HT (Gershon and Tack 2007). These are endoderm‐derived cells that express the non‐neuronal isoform of tryptophan hydroxylase, TPH1 (Walther et al. 2003), which synthesizes 5‐HT for subsequent packaging into vesicles. 5‐HT secretion from primary EC cells results from membrane depolarization triggering the entry of extracellular Ca^2+^ through L‐type voltage‐gated Ca^2+^ channels (Raghupathi et al. 2013; Zelkas et al. 2015). Much research has focused on roles of gut‐derived 5‐HT within the GI tract, including our own work proving that GI motility is regulated by gut‐derived 5‐HT but can be maintained without it (Keating and Spencer 2010; Spencer et al. 2011).

Studies in mucosal tissue from the human colon, immortalized cell line models of EC cells (BON cells), and human primary colonic EC cell cultures demonstrate that 5‐HT is released in response to a variety of stimuli (Modlin et al. 2006; Kidd et al. 2008; Symonds et al. 2015), including nutrients. EC cells are exposed to ingested nutrients, bile acids, and signals produced by gut microflora, as well as to circulating nutrients. Each nutrient source may have distinct effects on EC cell signaling outputs. For example, luminal glucose triggers 5‐HT release from guinea pig primary colonic EC cells and intact mouse tissue preparations (Zelkas et al. 2015). The sodium‐dependent glucose transporters SGLT1 and SGLT3 have each been implicated in driving membrane depolarization in response to increased glucose in BON cells and rodent intestine, respectively (Kim et al. 2001; Freeman et al. 2006).

Dietary nutrients present in the GI tract also change throughout its length. For example, hexoses such as glucose and fructose are preferentially detected and absorbed in the proximal intestine for use in energy metabolism. Luminal glucosidase enzymes hydrolyze more complex sugars, a process which has been associated with reduced feeding behavior via the 5‐HT_3_ receptor (Savastano et al. 2005b). Under healthy conditions, absorption of luminal sugars is largely complete by the time ingested contents have reached the colon. Additionally, GI microbiota, found primarily in the colon, are capable of producing their own nutrient sources including short‐chain fatty acids (SCFAs). SCFAs have recently been shown to enhance 5‐HT synthesis in EC cells (Fukumoto et al. 2003; Alemi et al. 2013; Nzakizwanayo et al. 2015; Yano et al. 2015), possibly through activation of the free fatty acid receptor 2 (FFAR2) (Akiba et al. 2015). Given that the types of nutrients and their respective concentrations differ along the GI tract, the mechanisms involved in sensing these nutrients may also be dependent on location within the GI tract. To understand this further, we have isolated primary EC cells from the duodenum and colon of mice to undertake comparative studies of responses to specific nutrient types.

## Methods

### Primary mouse EC cell isolation

Animal studies were performed in accordance with the guidelines of the Animal Ethics Committee of Flinders University. Male, 8‐ to 16‐week‐old C57BL/6 mice fed a standard chow diet were humanely killed by isoflurane overdose and cervical dislocation. Duodenum and colon were immediately removed and EC cells isolated and purified according to our previously published methods (Raghupathi et al. 2013; Martin et al. 2017). In brief, the mucosal layer was removed in 4°C Krebs buffer (in mmol/L; NaCl 140, KCl 5, CaCl_2_ 2, MgCl_2_ 1, HEPES 10, d‐glucose 5, pH 7.4), minced, and digested in a combination of 1 part collagenase A (3 mg mL^−1^, Roche Diagnostics GmbH, Mannheim, Germany) and 2 parts 0.05% trypsin‐EDTA (Sigma‐Aldrich, St. Louis, MO, USA) at 37°C for 30–40 min with constant agitation. Tissue digestion was inactivated by addition of equal volumes of DMEM culture media (Gibco, Grand Island, NY, USA) containing 10% FBS, 1% l‐glutamine, and 1% penicillin–streptomycin, and the digestion mixture was filtered through a 40‐*μ*m cell strainer (Grainer Bio‐One, Monroe, NC, USA) and centrifuged at 600*g*. The supernatant was removed and the resulting pellet resuspended in 1 mL of culture media, which was then layered on top of a Percoll (Sigma‐Aldrich) density gradient formed according to the manufacturer's instructions. Following centrifugation at 1100*g* for 8 min and slow braking, EC cells were harvested at a Percoll density of 1.059–1.07 g L^−1^. Harvested cells were washed once, then resuspended in culture media. EC cell viability was measured by Trypan blue staining (0.2% final concentration) followed by cell counting using a hemocytometer. Cells were considered viable if they completely excluded the dye. The purity of EC cell‐isolated cultures was determined by immunofluorescence staining for 5‐HT and TPH1, as per our previously described methods (Raghupathi et al. 2013, 2016; Nzakizwanayo et al. 2015; Martin et al. 2017).

### Ca^2+^ flux by flow cytometry

Isolated EC cells were centrifuged at 500*g* for 4 min, then resuspended in 1 mL of Hank's balanced salt solution (HBSS, Sigma‐Aldrich) supplemented with 1 mmol/L Ca^2+^ and 20 mmol/L HEPES. EC cells were incubated at 37°C for 35 min in the presence of the Ca^2+^ indicators, Fluo‐3 and Fura Red, washed once with HBSS, centrifuged at 450*g* for 4 min, then resuspended in HBSS at a final volume of 150 *μ*L per FACS tube, with 1.0–3.0 × 10^5^ cells per tube. Intracellular Ca^2+^ (Ca^2+^_(i)_) flux was determined using a BD FACSCanto II (BD Biosciences) flow cytometer. Following a 10‐sec baseline recording, a stimulation solution containing 100–500 mmol/L of one of the hexoses: glucose, fructose, sucrose, *α*‐MG, or 1–100 mmol/L of the SCFAs: acetate, butyrate, or propionate, was added and recording continued for a further 150 sec. The concentrations of hexoses were chosen based on luminal glucose concentrations within the GI tract having been proposed to reach 300 mmol/L at the brush‐border membrane (BBM) following a meal (Pappenheimer 1993), while the sucrose concentration of a standard sugar‐sweetened beverage can exceed 600 mmol/L. In colon preparations, however, the concentration of the different sugar stimulants did not exceed 300 mmol/L, as concentrations higher than this significantly decreased cell viability. SCFA concentrations were based on the concentrations reported in the lumen of the colon under healthy conditions (Cummings et al. 1987; Yajima and Sakata 1992; Topping and Clifton 2001; Alex et al. 2013). The Ca^2+^‐ATPase blocker, thapsigargin (TG), was used as a positive control to test the ability of cells to respond and for proper Ca^2+^ indicator loading. Data were expressed as the relative change in the ratio of Fluo‐3/Fura Red over time using FlowJo V10 (LLC, USA) for analyses. Changes in Fluo‐3/Fura Red were compared to the baseline time period for each recording, and compared to responses in unstimulated conditions (control). Baseline subtracted net area under the curve (AUC) was quantified using GraphPad PRISM 5.04 software.

### 5‐HT secretion by ELISA

Isolated EC cells were suspended in prewarmed Krebs solution containing 5‐HT stabilizer buffer (Labor Diagnosticka Nord) and 1 *μ*mol/L fluoxetine, to block potential 5‐HT reuptake via SERT and reduce potential metabolism of 5‐HT, and added to a 96‐well plate at a density of 1.5–2 × 10^4^ cells in 120 *μ*L per well. Cells were incubated to adhere and equilibrate for 30 min at 37°C, 5% CO_2_. From each well, half the volume was collected (representing 0 min) and replaced with an equal volume of stimulation solution at two times concentration to achieve the desired assay concentration. To determine the minimum incubation time for detectable changes in 5‐HT concentration using 5‐HT ELISA, a time course was determined with exposure to glucose, sampling at 2, 5, 10, 20, and 120 min intervals. Since 20 min was the minimum incubation time for detectable changes in 5‐HT secretion by ELISA, we used this time frame as the bases for all hexose stimulation experiments. Forty *μ*L of solution was collected from each well after 20 min exposure to hexose stimulants, and 2 h exposure to SCFAs, placed on ice, and immediately stored at −20°C until further use. 5‐HT content of each sample was measured using a 5‐HT ELISA kit (BA E‐5900, BioStrategy) according to the manufacturer's instructions. Preliminary experiments were performed to confirm that addition of stimuli to supernatant in the absence of any cells does not influence the results of this ELISA (data not shown). Data are expressed as net 5‐HT secretion since 0 min, normalized to cell number.

## Results

### Glucose

**Figure 1:**
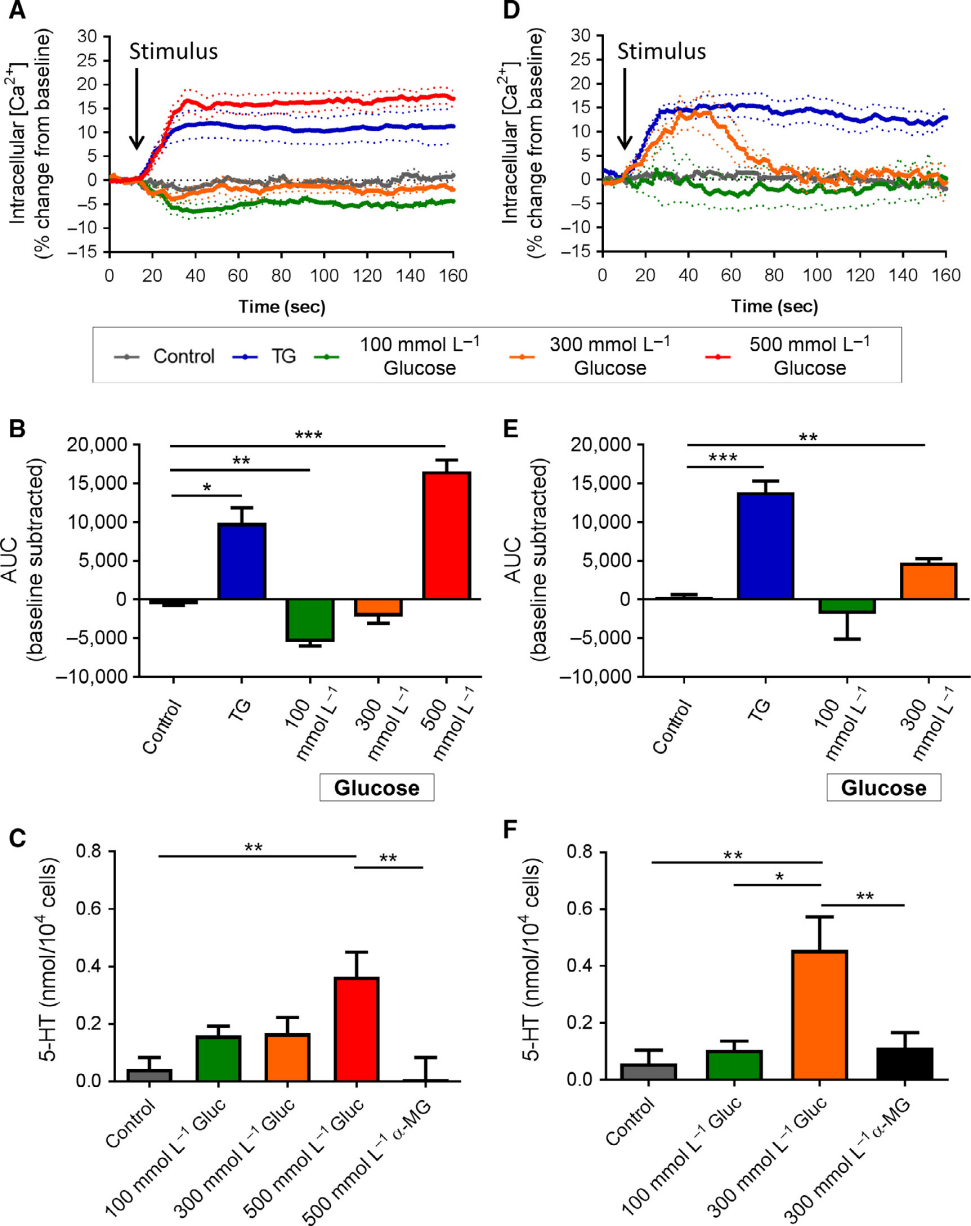
Effect of acute glucose stimulation on duodenal and colonic EC cells. (A) Time course of Ca^2+^_(i)_ changes in duodenal EC cells in response to glucose, shown as % change from baseline. Dotted lines indicate SEM. Arrow indicates time of stimulus addition (*n *=* *3–8 mice). TG, thapsigargin. (B) AUC of Ca^2+^_(i)_ in duodenal EC cells. *P <* *0.05, **P <* *0.01, ***P <* *0.001 (*n *=* *3–8 mice). (C) Release of 5‐HT from duodenal EC cells in culture following 20 min exposure to glucose (Gluc) or *α*‐MG. *P <* *0.01 (*n *=* *4–7 mice). (D) Time course of Ca^2+^_(i)_ changes in colonic EC cells in response to glucose, shown as % change from baseline. Arrow indicates time of stimulus addition (*n *=* *4–6 mice). (E) AUC of Ca^2+^_(i)_ in colonic EC cells. **P <* *0.01, ***P <* *0.001 (*n *=* *4–6 mice). (F) Release of 5‐HT from colonic EC cells in culture following 20 min exposure to glucose (Gluc) or *α*‐MG. *P <* *0.05, **P <* *0.01 (*n *=* *6–7 mice). Data are shown as mean ± SEM. AUC, area under the curve.

Calcium flux is a rapid and sensitive measure of cell activation. We monitored Ca^2+^ flux by flow cytometry by preloading the cells with the Ca^2+^ indicators Fluo‐3 and Fura red. These sensors increase and decrease fluorescence upon exposure to Ca^2+^, respectively, allowing a ratiometric analysis to be performed (Dustin 2000). The Fluo‐3/Fura red ratio was used to represent a comparable unit of intracellular Ca^2+^ level (Ca^2+^_(i)_), while net AUC (prestimulation baseline subtracted area under the curve) represented a measure of total net Ca^2+^ flux within the stimulation time period. For unstimulated control cells (in 5 mmol/L glucose), Ca^2+^_(i)_ and AUC were relatively unchanged across the 150 sec time period (Fig 1A and B). Stimulation of duodenal EC cells with 100 mmol/L glucose caused a sustained reduction in Ca^2+^_(i)_ compared to prestimulation baseline (Fig 1A), resulting in a negative total net flux compared to the unstimulated control (AUC *P *<* *0.01, Fig. 1B). No change in Ca^2+^_(i)_ from baseline, or change in AUC compared to unstimulated (5 mmol/L) control, was observed in response to 300 mmol/L glucose. However, 500 mmol/L glucose increased Ca^2+^_(i)_ (*P *<* *0.001 vs. control, Fig.  3A and B), exceeding that observed with positive stimulatory control, TG (*P *<* *0.05 vs. control). In order to relate these findings to 5‐HT secretion, 5‐HT release was determined by immunoassay. 5‐HT release from duodenal EC cells increased 20 min after exposure to 500 mmol/L glucose (0.36 ± 0.09 nmol/10^4^ cells), in comparison to 500 mmol/L *α*‐MG (0.01 ± 0.07 nmol/10^4^ cells, *P *<* *0.01) or 5 mmol/L glucose exposure (control group, 0.04 ± 0.04 nmol/10^4^ cells, *P* < 0.01, Fig. 1C).

In colonic cells, exposure to 100 mmol/L glucose had no effect on Ca^2+^_(i)_ levels. However, 300 mmol/L glucose transiently raised Ca^2+^_(i)_ (Fig 1D) resulting in increased total net Ca^2+^ flux (AUC *P *<* *0.01 vs. control, Fig. 1E). Release of 5‐HT from colonic EC cell in 20 min was increased when exposed to 300 mmol/L glucose (0.46 ± 0.12 nmol/10^4^ cells), in comparison to 100 mmol/L glucose (0.11 ± 0.03 nmol/10^4^ cells, *P *<* *0.01), to 300 mmol/L of the nonmetabolizable sugar *α*‐MG (0.11 ± 0.05 nmol/10^4^ cells, *P *<* *0.01) or to the 5 mmol/L glucose control (0.06 ± 0.05 nmol/10^4^ cells, *P *<* *0.01, Fig. 1F).

### Fructose

**Figure 2:**
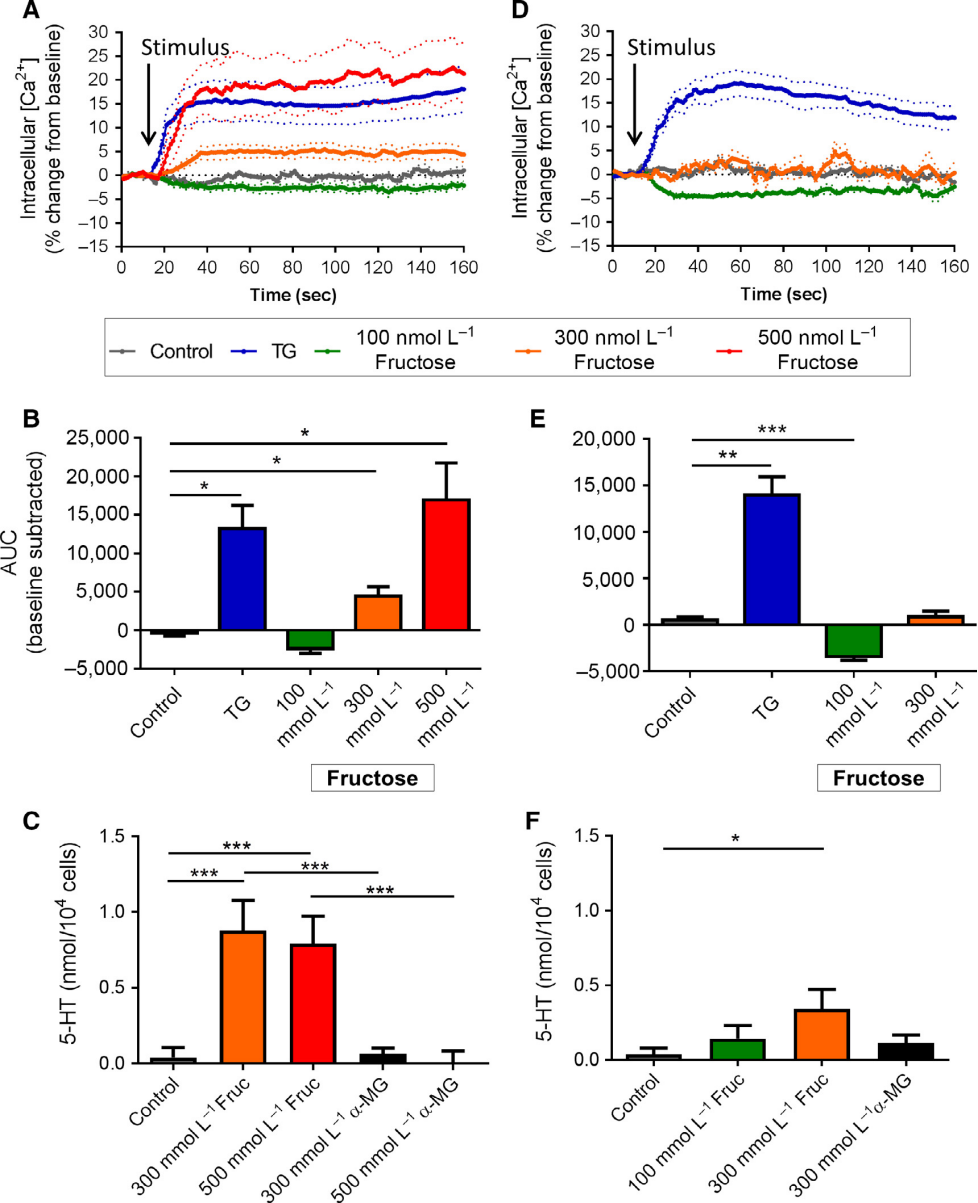
Effect of acute fructose stimulation on duodenal and colonic EC cells. (A) Time course of Ca^2+^_(i)_ changes in duodenal EC cells in response to fructose, shown as % change from baseline. Dotted lines indicate SEM. Arrow indicates time of stimulus addition (*n *=* *3–9 mice). (B) AUC of Ca^2+^_(i)_ in duodenal EC cells. *P <* *0.05 (*n *=* *3–9 mice). (C) Release of 5‐HT from duodenal EC cells in culture following 20 min exposure to fructose (Fruc) or *α*‐MG. ***P <* *0.001 (*n *=* *4–9 mice). (D) Time course of Ca^2+^_(i)_ changes in colonic EC cells in response to fructose, shown as % change from baseline. Arrow indicates time of stimulus addition (*n *=* *4–8 mice). (E) AUC of Ca^2+^_(i)_ in colonic EC cells. ***P <* *0.001 (*n *=* *4–8 mice). (F) Release of 5‐HT from colonic EC cells in culture following 20 min exposure to fructose (Fruc) or *α*‐MG. *P <* *0.05 (*n *=* *3–9 mice). Data are shown as mean ± SEM. AUC, area under the curve.

Exposure of duodenal EC cells to 100 mmol/L fructose did not change Ca^2+^_(i)_. However, dose‐dependent increases in Ca^2+^_(i)_ were observed in response to 300 mmol/L (AUC *P *<* *0.05 vs. control) and 500 mmol/L fructose (AUC *P *<* *0.05 vs. control), with the latter response comparable to that seen with TG (*P *<* *0.01 vs. control, Fig. 2A and B). Fructose exposure also increased 5‐HT secretion from duodenal EC cells at both 300 mmol/L (0.88 ± 0.12 nmol/10^4^ cells, *P *<* *0.001 vs. control and *α*‐MG) and 500 mmol/L fructose (0.79 ± 0.18 nmol/10^4^ cells, *P *<* *0.001 vs. control and *α*‐MG). No change in 5‐HT secretion was seen with *α*‐MG exposure (Fig. 2C).

The Ca^2+^_(i)_ response to fructose in colonic EC cells differed to that in duodenal EC cells (Fig. 2D). Exposure to 100 mmol/L fructose decreased Ca^2+^_(i)_ in colonic EC cells (AUC *P *<* *0.001 vs. control), but Ca^2+^_(i)_ did not change in the presence of 300 mmol/L fructose (Fig. 2E), as occurred in duodenal EC cells. 5‐HT secretion, however, was triggered from colonic EC cells after 20 min exposure to 300 mmol/L fructose (0.34 ± 0.13 nmol/10^4^ cells, *P *<* *0.01 vs. control, Fig. 2F).

### Sucrose

**Figure 3:**
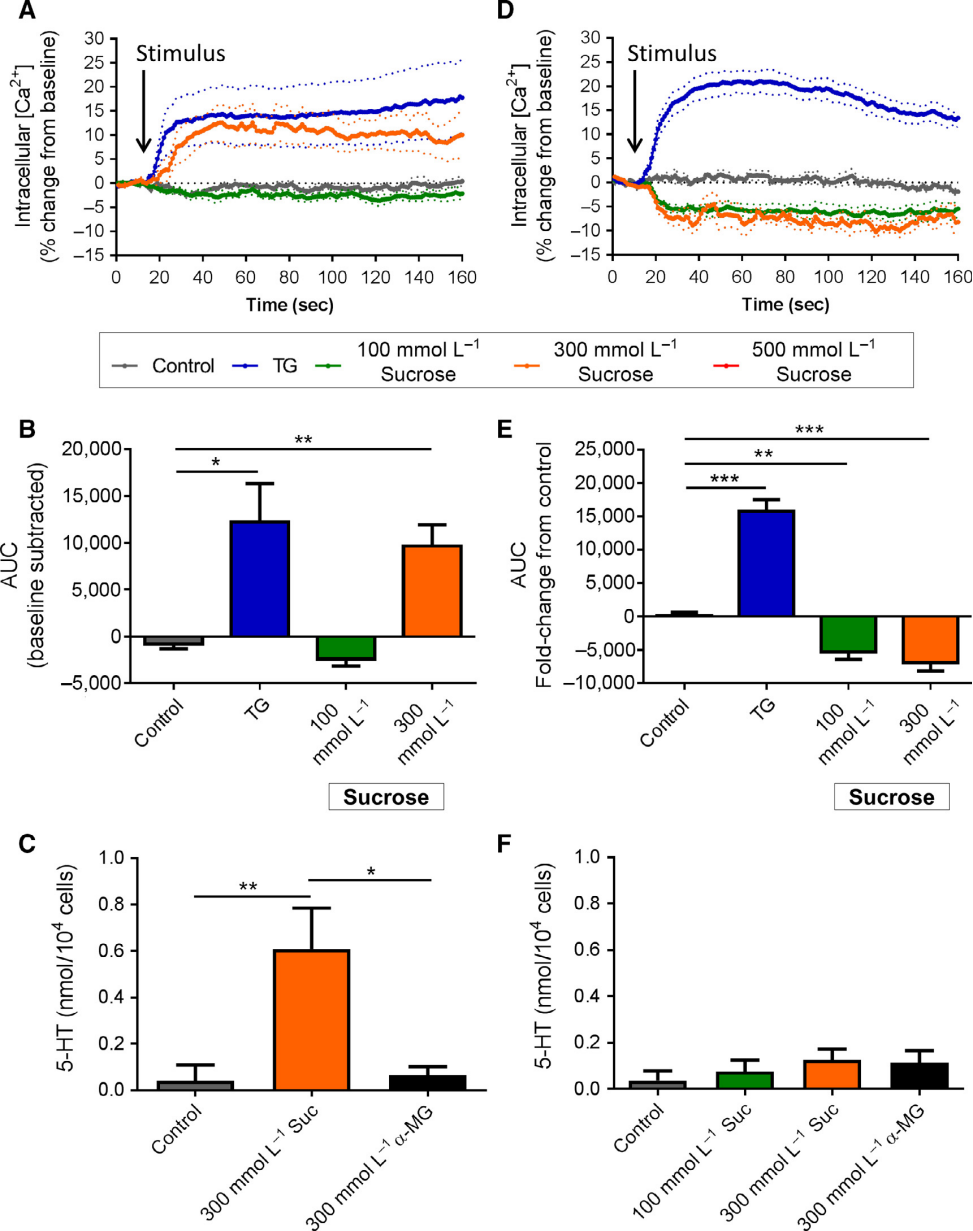
Effect of acute sucrose stimulation on duodenal and colonic EC cells. (A) Time course of Ca^2+^_(i)_ changes in duodenal EC cells in response to sucrose, shown as % change from baseline. Dotted lines indicate SEM. Arrow indicates time of stimulus addition (*n *=* *4–5 mice). (B) AUC of Ca^2+^_(i)_ in duodenal EC cells. *P <* *0.05, **P <* *0.01 (*n *=* *4–5 mice). (C) Release of 5‐HT from duodenal EC cells in culture following 20 min exposure to sucrose (Suc) or *α*‐MG. *P <* *0.05, **P <* *0.01 (*n *=* *6–11 mice). (D) Time course of Ca^2+^_(i)_ changes in colonic EC cells in response to sucrose, shown as % change from baseline. Arrow indicates time of stimulus addition (*n *=* *5 mice). (E) AUC of Ca^2+^_(i)_ in colonic EC cells. **P <* *0.01, ***P <* *0.001 (*n *=* *5 mice). (F) Release of 5‐HT from colonic EC cells in culture following 20 min exposure to sucrose (Suc) or *α*‐MG (*n *=* *3–9 mice). Data are shown as mean ± SEM. AUC, area under the curve.

Exposure of duodenal EC cells to 300 mmol/L sucrose induced a rapid and sustained increase in Ca^2+^_(i)_ (AUC *P *<* *0.01 vs. control), comparable with that seen with TG (*P* < 0.01 vs. control, Fig. 3A and B). An increase in 5‐HT secretion from duodenal EC cells was also seen with exposure to 300 mmol/L sucrose (0.61 ± 0.18 nmol/10^4^ cells, *P *<* *0.01 vs. control), which did not occur with exposure to 300 mmol/L *α*‐MG (*P *<* *0.05 vs. 300 mmol/L sucrose, Fig. 3C).

A decrease in Ca^2+^_(i)_ was observed in colonic EC cells in response to both 100 mmol/L sucrose (*P *<* *0.01 vs. control) and 300 mmol/L sucrose (*P *<* *0.001 vs. control) (Fig. 3D and E). No increase in 5‐HT secretion was observed at any of the sucrose concentrations (Fig. 3F).

### SCFA

**Figure 4:**
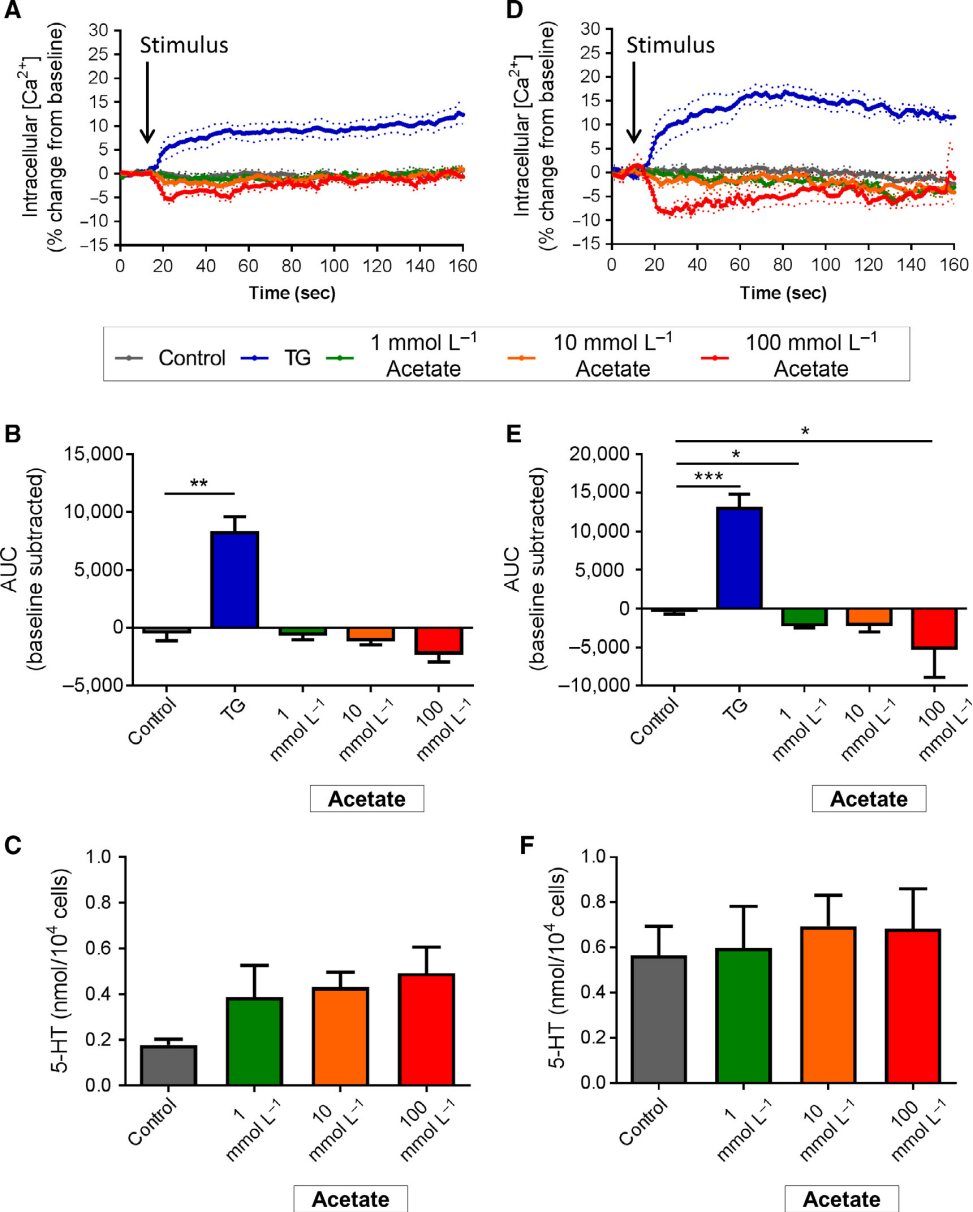
Effect of acetate stimulation on duodenal and colonic EC cells. (A) Time course of Ca^2+^_(i)_ changes in duodenal EC cells in response to acetate, shown as % change from baseline. Dotted lines indicate SEM. Arrow indicates time of stimulus addition (*n *=* *4–5 mice). (B) AUC of Ca^2+^_(i)_ in duodenal EC cells. ^**^ *P *<* *0.01 (*n *=* *5 mice). (C) Release of 5‐HT from duodenal EC cells in culture following 2 h exposure to acetate (Acet) or *α*‐MG. (*n *=* *5–6 mice). (D) Time course of Ca^2+^_(i)_ changes in colonic EC cells in response to sucrose, shown as % change from baseline. Arrow indicates time of stimulus addition (*n *=* *4–7 mice). (E) AUC of Ca^2+^_(i)_ in colonic EC cells. *P <* *0.05, ***P <* *0.001 (*n *=* *4–7 mice). (F) Release of 5‐HT from colonic EC cells in culture following 2 h exposure to acetate (*n *=* *5–8 mice). Data are shown as mean ± SEM. AUC, area under the curve.

EC cells were tested for response to the SCFAs acetate, butyrate, and propionate. Duodenal EC cells did not change Ca^2+^_(i)_ or secrete 5‐HT in response to a range of acetate concentrations from 1 to 100 mmol/L (Fig. 4A–C). Ca^2+^_(i)_ was reduced in colonic EC cells in response to 1 mmol/L (*P *<* *0.05 vs. control) and 100 mmol/L acetate (*P *<* *0.05 vs. control, Fig. 4D and E), however, this was not associated with any changes in 5‐HT secretion (Fig. 4F).

**Figure 5:**
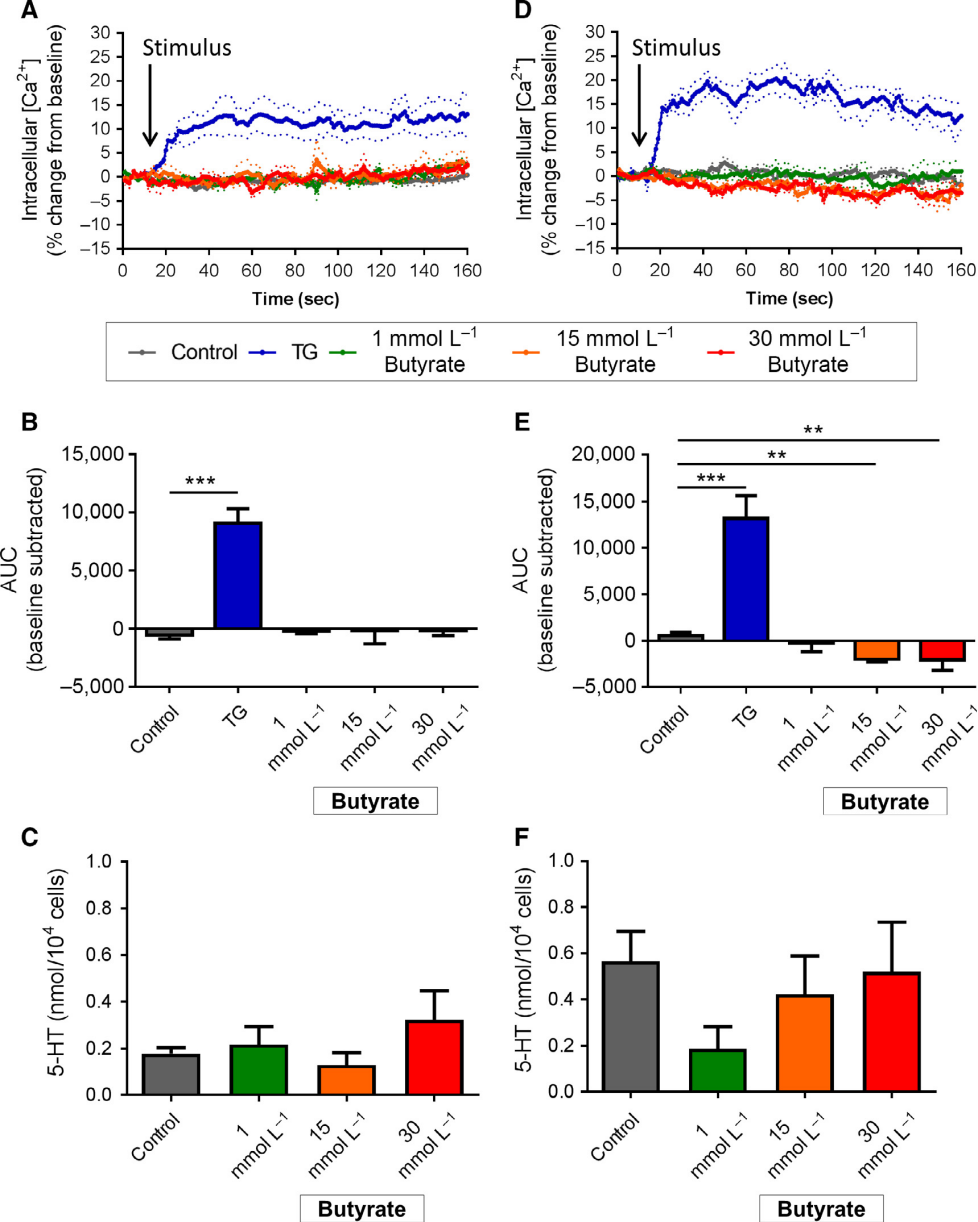
Effect of butyrate stimulation on duodenal and colonic EC cells. (A) Time course of Ca^2+^_(i)_ changes in duodenal EC cells in response to butyrate, shown as % change from baseline. Dotted lines indicate SEM. Arrow indicates time of stimulus addition (*n *=* *4–5 mice). (B) AUC of Ca^2+^_(i)_ in duodenal EC cells. ***P <* *0.001 (*n *=* *4–5 mice). (C) Release of 5‐HT from duodenal EC cells in culture following 2 h exposure to butyrate (But) or *α*‐MG (*n *=* *5 mice). (D) Time course of Ca^2+^_(i)_ changes in colonic EC cells in response to butyrate, shown as % change from baseline. Arrow indicates time of stimulus addition (*n *=* *3–4 mice). (E) AUC of Ca^2+^_(i)_ in colonic EC cells. **P <* *0.01 (*n *=* *3–4 mice). (F) Release of 5‐HT from colonic EC cells in culture following 2 h exposure to butyrate. (*n *=* *5–7 mice). Data are shown as mean ± SEM. AUC, area under the curve

Exposure to increasing concentrations of butyrate from 1 to 30 mmol/L did not alter Ca^2+^_(i)_ or release 5‐HT from duodenal EC cells (Fig. 5A–C). In colonic EC cells, however, Ca^2+^_(i)_ was reduced after exposure to both 15 mmol/L (*P* < 0.05 vs. control, Fig 5D) and 30 mmol/L butyrate (*P* < 0.05 vs. control, Fig. 5E). No change in 5‐HT secretion was observed after 2 hr incubation with butyrate (Fig. 5F).

**Figure 6:**
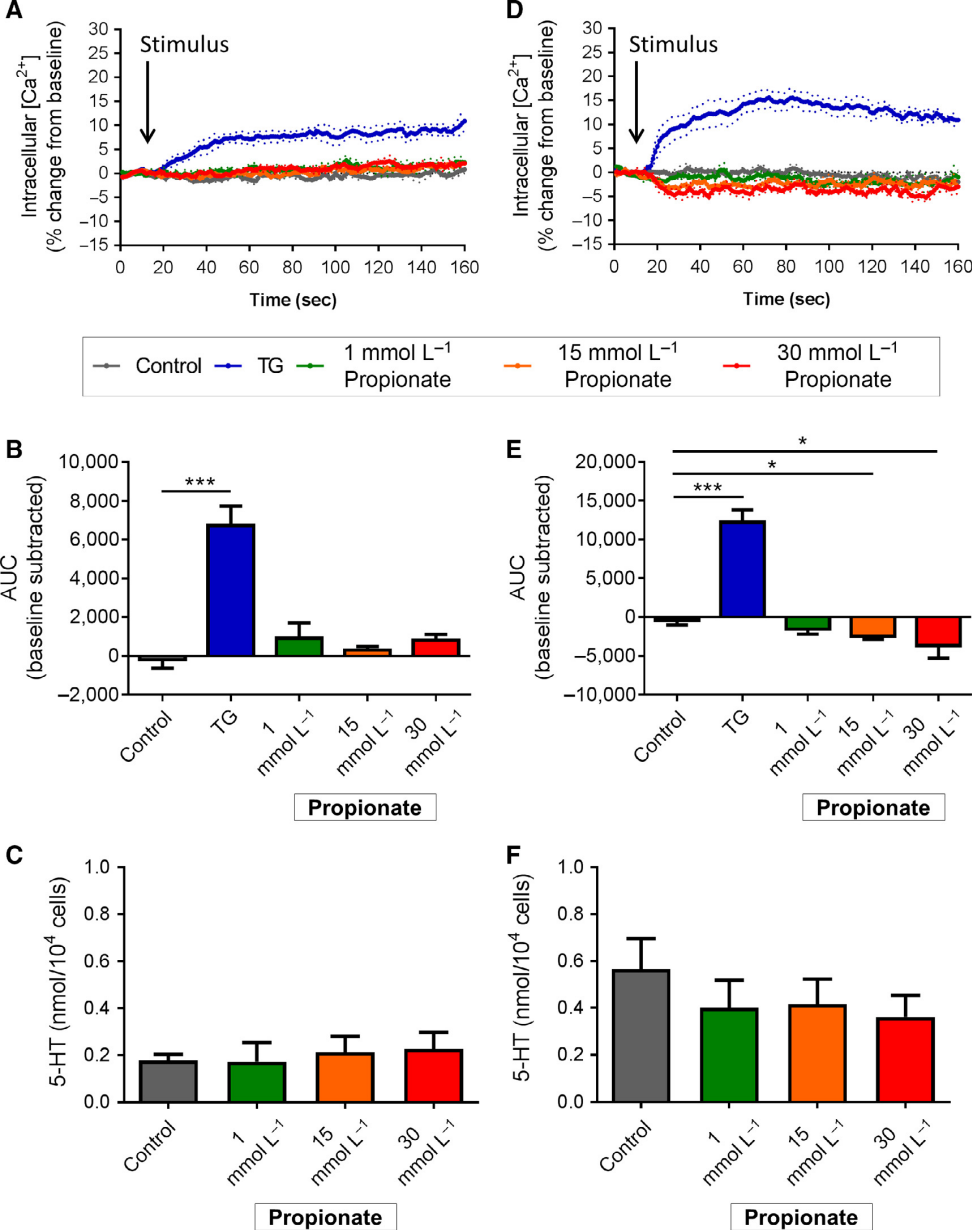
Effect of propionate stimulation on duodenal and colonic EC cells. (A) Time course of Ca^2+^_(i)_ changes in duodenal EC cells in response to propionate, shown as % change from baseline. Dotted lines indicate SEM. Arrow indicates time of stimulus addition (*n *=* *4 mice). (B) AUC of Ca^2+^_(i)_ in duodenal EC cells. *P <* *0.05, ***P <* *0.001 (*n *=* *4 mice). (C) Release of 5‐HT from duodenal EC cells in culture following 2 h exposure to propionate (Prop) or *α*‐MG (*n *=* *5 mice). (D) Time course of Ca^2+^_(i)_ changes in colonic EC cells in response to propionate, shown as % change from baseline. Arrow indicates time of stimulus addition (*n *=* *5 mice). (E) AUC of Ca^2+^_(i)_ in colonic EC cells. *P <* *0.05, ***P <* *0.001 (*n *=* *5 mice). (F) Release of 5‐HT from colonic EC cells in culture following 2 h exposure to propionate (*n *=* *5–6 mice). Data are shown as mean ± SEM. AUC, area under the curve.

Duodenal EC cells did not change Ca^2+^_(i)_ or secrete 5‐HT in response to increasing concentrations of propionate from 1 to 30 mmol/L (Fig. 6A–C). Colonic EC cells were unresponsive to 1 mmol/L propionate, however, Ca^2+^_(i)_ decreased upon exposure to 15 mmol/L (*P *<* *0.05 vs. control) and 30 mmol/L propionate (*P *<* *0.05 vs. control, Fig. 6D and E). This was not associated with any change in 5‐HT secretion after 2 h exposure to propionate (Fig. 6F).

## Discussion

This study compared differences in nutrient sensing capacity in primary mouse EC cells obtained from duodenum and colon of the same animal. This provides a powerful approach allowing for a paired comparison of nutrient responses in EC cells obtained from different regions of the GI tract. A major finding is our demonstration of region‐specific responses of duodenal and colonic EC cells to sugars, as evidenced by differential increases in Ca^2+^_(i)_ and 5‐HT release. In particular, colonic EC cells showed higher sensitivity to glucose, while duodenal EC cells were more sensitive to fructose and sucrose. While sugars were found to elicit secretion of gut 5‐HT, acute exposure to SCFAs did not. Importantly, this 5‐HT secretion was nutrient dependent and did not occur secondary to osmotic influences, as the nonmetabolizable glucose analog, *α*‐MG, did not increase 5‐HT secretion in either duodenal or colonic EC cells at effective sugar doses to 500 mmol/L. This provides strong support for a receptor‐ or transporter‐mediated response underlying nutrient‐induced 5‐HT secretion.

Our data demonstrate that mouse EC cells do not respond to glucose at levels seen in circulation, and respond only to levels that occur within the GI tract following nutrient ingestion. While the concentration of sugars used to stimulate cells in this study appear high, luminal glucose concentrations within the GI tract have been proposed to reach 300 mmol/L at the brush‐border membrane (BBM) following a meal (Pappenheimer 1993), whereas the sucrose concentration of a standard sugar‐sweetened beverage can exceed 600 mmol/L. The fact that we find duodenal 5‐HT secretion occurs in response to 500 mmol/L, but not 300 mmol/L glucose, is likely due to the tuning of EC cell sensing machinery to luminal glucose cues.

We found that duodenal EC cells were more responsive to fructose than colonic EC cells. Fructose triggered Ca^2+^ entry and 5‐HT release in duodenal cells at 300 and 500 mmol/L. Despite equivalent 5‐HT secretion, intracellular Ca^2+^ influx in response to 300 mmol/L fructose stimulation was, however, significantly lower compared to 500 mmol/L fructose stimulation. This may be due to the level of Ca^2+^ needed for peak 5‐HT secretion occurring at this concentration, with increased Ca^2+^ above this level causing no further increase in 5‐HT secretion. However, while 300 mmol/L fructose triggered a small increase in 5‐HT release in colonic EC cells, it did so in the absence of an increase in cellular Ca^2+^ levels. It is possible that this difference is a result of the different duration of experiments used to measure intracellular calcium and 5‐HT secretion, and that significant amounts of Ca^2+^ may enter the cell in response to fructose over 20 min. Such a long duration was not possible to measure with our Ca^2+^ imaging approach. Exposure to fructose for 20 min in culture could also have permitted translocation of GLUT2/GLUT5 transporters to the plasma membrane (Mace et al. 2007) to increase 5‐HT release in colonic EC cells, a process unlikely to be evident during the Ca^2+^ measurement time frame. Alternatively, fructose may trigger 5‐HT release from colonic EC cells via Ca^2+^‐independent mechanisms.

High sucrose concentrations triggered similar release of 5‐HT release from duodenal, but not colonic EC cells. However, in this case, 300 mmol/L sucrose did not trigger Ca^2+^ entry or 5‐HT release in colonic EC cells. Hexose sugars and sweeteners are detected by the taste receptor family of proteins, which form as either heterodimers or homodimers. While the T1R2/T1R3 receptor heterodimer is expressed in the duodenum of humans (Young et al. 2009), T1R2 has been immunolocalized to only a small subset of human duodenal EC cells (Young et al. 2013), and T1R2 gene expression has not been detected in EC cells (Kidd et al. 2008; Martin et al. 2017). The lack of T1R2 suggests that the ability of duodenal EC cells to sense sucrose may occur via a T1R3 receptor homodimer, which has been identified as a low‐affinity glucose sensor in pancreatic *β* cells (Kojima et al. 2015).

The decrease in intracellular Ca^2+^ levels observed following stimulus exposure in some preparations, with predominantly lower stimulant concentrations, was unexpected and cannot be explained with the current methodology. Removal of Ca^2+^ from the intracellular space may be due to the resequestering of Ca^2+^ to endoplasmic stores, or extracellular release of Ca^2+^ due to changes in membrane permeability or ion transport channels. It is plausible that this is in efforts to maintain Ca^2+^ homeostasis through clearance of intracellular Ca^2+^, a mechanism shown to terminate a stimulus response in isolated mouse taste receptor cells through Na^+^–Ca^2+^ exchange (Szebenyi et al. 2010).

Intraduodenal infusion of sugars slows gastric emptying and nutrient intake in rodents and humans, which occurs via an extrinsic nerve reflex which is triggered, in part, by stimulation of 5‐HT_3_ receptors on vagal sensory neurons (Rayner et al. 2000; Raybould et al. 2003; Savastano et al. 2005a). Release of 5‐HT from EC cells in response to luminal sugars appears central to this pathway. Such glucose‐induced release of 5‐HT also stimulates duodenal bicarbonate secretion (Tuo et al. 2004) and suppresses the uptake of sodium from the lumen, thus affecting luminal water and electrolyte absorption (Imada‐Shirakata et al. 1997; Gill et al. 2005). The polarity of 5‐HT secretion from EC cells in gut, in response to luminal cues, has not been established. However, cell polarity has been shown to play an important role in luminal nutrient sensing in L cells (Kuhre et al. 2015), with nutrient sensing receptors and transporters exhibiting polar expression on either brush‐border or basolateral membranes (Mace et al. 2007). Possible differences in nutrient sensing between the apical and basolateral membrane of EC cells cannot be established using our single‐cell preparations, in which the polarity of EC cells becomes lost. In addition, intercellular interactions within the native environment of the gut, which could potentially modulate the response to luminal nutrients, are also lost. How changes to cell polarity and environment affect the basic cellular responses of EC cells to nutrients in culture is unknown. However, our findings of glucose sensing in isolated colonic EC cells is consistent with our previous work using intact colonic tissue preparations, in which cell‐to‐cell interactions and cell polarity are maintained (Zelkas et al. 2015).

The ability of glucose to trigger colonic 5‐HT release suggests a role of colonic 5‐HT in GI disease, as this region would have limited exposure to luminal glucose under situations of normal GI transit. However, in disease states with reduced intestinal transit time, the presence of higher luminal concentrations of ingested sugars may occur in the colon. It is possible that the increased sensitivity of colonic EC cells to glucose may play a role in side effects often observed in patients following Roux‐en‐Y gastric bypass surgery, which include diarrhea and nausea, and the often occurring complication of dumping syndrome (Tack and Deloose 2014). Nutrient‐induced 5‐HT release may also affect a number of systemic physiological processes, particularly those involved in energy metabolism. Peripheral 5‐HT release has been recently shown to augment hepatic gluconeogenesis to increase fasting blood glucose levels (Sumara et al. 2012), and to inhibit thermogenesis in brown adipose tissue (Crane et al. 2015). In addition, sweet taste triggered 5‐HT release from duodenal EC cells may also be important in the setting of metabolic disorders such as obesity and type 2 diabetes, where there is defective regulation of intestinal sweet taste receptors and exaggerated postprandial glucose absorption (Young et al. 2013).

Our finding that SCFAs do not acutely stimulate EC cell 5‐HT secretion is, perhaps, not surprising, given the sources of SCFAs available to these cells is likely low level constant exposure from gut microbiota. Gut microbiota are the primary source of these nutrients within the gut, especially in the colon which contains the largest source of gut bacteria (Donaldson et al. 2016). We chose a 2‐h incubation time for these experiments as this was adequate to observe a response to SCFAs in colonic L cells (Chambers et al. 2015). Our data demonstrate that SCFAs do not induce a direct secretory effect in mouse EC cells. This is in contrast to the established SCFA‐dependent increases in *Tph1* expression and 5‐HT synthesis in EC cells observed with longer exposure of at least 4 h (Essien et al. 2013; Reigstad et al. 2015; Yano et al. 2015). What drives increases in *Tph1* expression in response to these fatty acids is unknown, but constitutes an important potential link between gut microbiome and host physiology.

Our study provides the first comparative analysis of acute nutrient sensing capacity of EC cells from the mouse duodenum and colon. It is also the first to demonstrate the capacity for isolated primary EC cells from mice to respond to nutrients. This approach has revealed that duodenal EC cells are more responsive than colonic EC cells to fructose and sucrose, while the opposite is true for glucose responses. It has also revealed that SCFAs do not trigger acute Ca^2+^ entry or 5‐HT secretion at either intestinal site. Correspondingly, the responsiveness of intestinal EC cells to ingested nutrients is likely to be diverse and region dependent.

## Conflict of Interest

The authors declare no conflicts of interest in relation to this research.
